# *In Vitro* Antioxidant and Cytotoxic Activities of *Arnebia benthamii* (Wall ex. G. Don): A Critically Endangered Medicinal Plant of Kashmir Valley

**DOI:** 10.1155/2014/792574

**Published:** 2014-03-06

**Authors:** Showkat Ahmad Ganie, Tanveer Ali Dar, Rabia Hamid, Ovais Zargar, Shayaq Ul Abeer, Akbar Masood, Shajrul Amin, Mohammad Afzal Zargar

**Affiliations:** ^1^Clinical Biochemistry, University of Kashmir, Hazratbal, Srinagar 190006, India; ^2^Department of Biochemistry, University of Kashmir, Hazratbal, Srinagar 190006, India; ^3^Department of Biotechnology, University of Kashmir, Srinagar, Hazratbal, Srinagar 190006, India

## Abstract

*Arnebia benthamii* is a major ingredient of the commercial drug available under the name Gaozaban, which has antibacterial, antifungal, anti-inflammatory, and wound-healing properties. In the present study, *in vitro* antioxidant and anticancer activity of different extracts of *Arnebia benthamii* were investigated. Antioxidant potential of plant extracts was evaluated by means of total phenolics, DPPH, reducing power, microsomal lipid peroxidation, and hydroxyl radical scavenging activity. The highest phenolic content (TPC) of 780 mg GAE/g was observed in ethyl acetate, while the lowest TPC of 462 mg GAE/g was achieved in aqueous extract. At concentration of 700 µg/mL, DPPH radical scavenging activity was found to be highest in ethyl acetate extract (87.99%) and lowest in aqueous extract (73%). The reducing power of extracts increased in a concentration dependent manner. We also observed its inhibition on Fe^2+^/ascorbic acid-induced lipid peroxidation (LPO) on rat liver microsomes *in vitro*. In addition, *Arnebia benthamii* extracts exhibited antioxidant effects on Calf thymus DNA damage induced by Fenton reaction. Cytotoxicity of the extracts (10–100 µg/mL) was tested on five human cancer cell lines (lung, prostate, leukemia, colon, and pancreatic cell lines) using the Sulphorhodamine B assay.

## 1. Introduction

Research on relationships between antioxidants and prevention of noncommunicable disease, such as cardiovascular disease, cancer, and diabetes has been increasing sharply in recent years. Free radicals have been claimed to play a key role in affecting human health by causing severe diseases, such as cancer and cardiovascular diseases by cell degeneration. These free radicals can be generated during normal body function and can be acquired from the environment. Oxygen radicals can cause damage to biomolecules (lipids, proteins, and DNA), eventually leading to many chronic diseases such as atherosclerosis, cancer, diabetics, rheumatoid arthritis, postischemic perfusion injury, myocardial infarction, cardiovascular diseases, chronic inflammation, stroke and septic shock, aging, and other degenerative diseases in humans [1]. Human body has multiple mechanisms especially enzymatic and nonenzymatic antioxidant systems to protect the cellular molecules against reactive oxygen species (ROS) induced damage [2]. However the innate defense may not be enough for severe or continued oxidative stress. Hence,certain amounts of exogenous antioxidants are constantly required to maintain an adequate level of antioxidants in order to balance the ROS in human body. There are several types of synthetic antioxidants such as butylated hydroxyanisole (BHA), butylated hydrotoluene (BHT), and tertiary-butylhydroquinone (TBHQ) which have been widely used in foods to prevent oxidation. The use of synthetic antioxidants in food, however, is discouraged because of their toxicity and carcinogenicity [3]. Hence, compounds especially from natural sources capable of protecting against ROS mediated damage may have potential application in prevention and/or curing of diseases.

*Arnebia benthamii *is a monocarpic perennial and reaches reproductive maturity in 3-4 years. The basal part of the root, leaves, and flowering stalk can be utilized for consumption and for trade.* Arnebia benthamii* is a major ingredient of the commercial drug available under the name Gaozaban, which has antifungal, anti-inflammatory, and wound-healing properties. The roots yield a red pigment, Shikonin (a dye), which has several medicinal properties and is marketed under the trade name Ratanjot and alkanin, a lipophilic red pigment which is the main active constituent of this plant and is responsible for its colour and therapeutic efficacy. On folklore levels the plant is used for curing various diseases of tongue, throat, fever, and cardiac disorders and has wound healing properties. The root has anthelmintic, antipyretic, and antiseptic property.* Arnebia benthamii* is used for imparting pleasing red colour to foodstuff, oils, and fats. The plant also possesses stimulant, tonic, diuretic, and expectorant properties. The flowering shoots are used in preparation of sherbet (syrup) and jam useful in various diseases of tongue, throat, fever, and cardiac disorders. No work has been done so far on this endemic plant of Kashmir valley to determine its antioxidant and antibacterial activities. The main objective of this preliminary investigation was to evaluate the protective effects of different extracts of* Arnebia benthamii* against free radical mediated damages under* in vitro* situations.* In vitro* assays were carried on DPPH radical scavenging activity, total phenolic content (TPC), reducing power, microsomal lipid peroxidation, hydroxyl radical scavenging activity, and Calf thymus DNA damage. In addition, the anticancer effect of different extracts on five human cancer cell lines was also investigated by Sulphorhodamine B (SRB) assay. Results from this study provide a better understanding of the nutritional and health benefits of this medicinal plant of Kashmir valley.

## 2. Materials and Methods

### 2.1. Chemicals

1, 1-Diphenyl-2-picrylhydrazyl (DPPH), gallic acid, Folin-Ciocalteu reagent, ascorbic acid, and SRB were purchased from Sigma-Aldrich. All other chemicals were of analytical grade and obtained from Himedia Company.

### 2.2. Plant Material

The* Arnebia benthamii* was collected from higher altitudes of Gulmarg, Jammu, and Kashmir state, India, in the months of September and October 2012, identified by the Centre of Plant Taxonomy, Department of Botany, University of Kashmir, and authenticated by Dr. Irshad Ahmad Nawchoo (Department of Botany) and Mr. Akhter Hussain Malik (Curator, Centre for Plant Taxonomy, University of Kashmir). A reference specimen has been retained in the herbarium of the Department of Botany at the University of Kashmir under reference number KASH-bot/Ku/AB-702-SAG.

### 2.3. Extract Preparation

**Figure 1:**
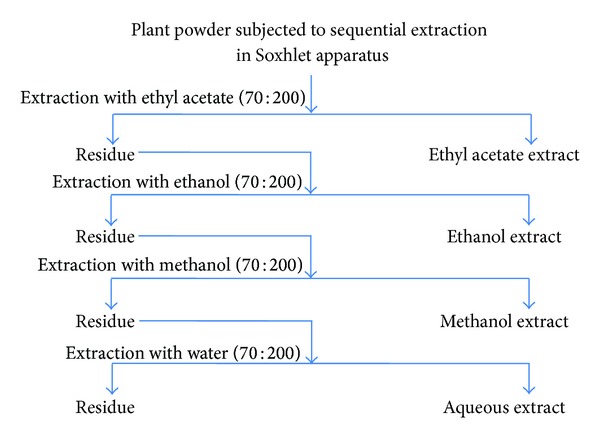
Systematic representation of preparation of different solvent extracts of* Arnebia benthamii* by sequential extraction method.

The whole plant material was dried in the shade at 30 ± 2°C. The dried material was ground into a powder using mortar and pestle and passed through a sieve of 0.3 mm mesh size. The powder obtained was extracted with different solvents like methanol, ethanol, ethyl acetate, and water for 48 hrs using a Soxhlet extractor (60–80°C) (Figure 1). The extract was then concentrated with the help of rotary evaporator under reduced pressure and the solid extract was stored in refrigerator for further use.

### 2.4. Determination of Total Phenolic Content

The TPC of the extracts of* Arnebia benthamii* was measured by the Folin-Ciocalteu method described with some modifications [4]. Briefly, an aliquot of 0.5 mL of sample solution (with appropriate dilution to obtain absorbance in the range of the prepared calibration curve) was mixed with 1.0 mL Folin-Ciocalteu reagent (10 times dilution before use) and allowed to react at 30°C for 5 min in the dark. Then 2.0 mL of saturated Na_2_CO_3_ solution was added and the mixture was allowed to stand for 1 h before the absorbance of the reaction mixture was read at 747 nm. A calibration curve, using gallic acid with a concentration range of 0.01–0.10 mg/mL, was prepared. The TPC of the samples was standardized against gallic acid and expressed as mg gallic acid equivalent (GAE) per gram of sample on a dry weight basis.

### 2.5. DPPH Radical Scavenging Activity

DPPH method was carried out according to the method modified by Kim et al. [5]. An aliquot of the radical formed from DPPH was left to react with 100–700 *μ*g/mL of the extract for 30 min. The absorbance was read at 517 nm. Catechin was used as the standard (10 mg/10 mL). The percentage of radical inhibition was calculated by the following formula:

(1) $$\begin{array}{c} \%\;\text{inhibition}=\left[1-\left(\frac{A_{e}}{A_{0}}\right)\right]\times 100, \end{array}$$

where *A*_0_ is the absorbance without sample, and *A*_*e*_ is absorbance with sample.

### 2.6. Reducing Power Test

The reducing power test based on Fe (III) to Fe (II) transformation in the presence of the solvent fractions was carried out by using the method of Oyaizu [6]. The Fe (II) can be monitored by measuring the formation of Perl's Prussian blue at 700 nm. Various concentrations of the sample (2 mL) were mixed with 2 mL of phosphate buffer (0.2 M, pH 6.6) and 2 mL of potassium ferricyanide (10 mg/mL). The mixture was incubated at 50°C for 20 min followed by addition of 2 mL of trichloroacetic acid (100 mg/L). The mixture was centrifuged at 1500 ×g for 10 min to collect the upper layer of the solution. A volume of 2 mL from each of the mixture earlier mentioned was mixed with 2 mL of distilled water and 0.4 mL of 0.1% (w/v) fresh ferric chloride. After 10 min reaction, the absorbance was measured at 700 nm. Higher absorbance of the reaction mixture indicates a higher reducing power.

### 2.7. Microsomal Lipid Peroxidation

Liver was washed in ice cold 1.15% KCl and homogenized in a homogenizing buffer (50 mM Tris-HCl, 1.15% KCl pH 7.4) using Teflon homogenizer. The homogenate was centrifuged at 9,000 ×g for 20 minutes to remove debris. The supernatant so obtained was further centrifuged at 15,000 rpm for 20 minutes at 4°C to get post mitochondrial supernatant (PMS). Microsomes were obtained by centrifuging the portion of prepared PMS by using Sorvall Ultracentrifuge at 105,000 ×g for 1 hr at 4°C to obtain the microsomal fraction. This fraction was resuspended in 0.25 M sucrose and stored frozen until use.

Rat liver microsomal lipid peroxidation was carried out according to the method of Urata et al. [7] with little modifications. The test sample (20–100 *μ*g/mL) was added to 1 mL of liver microsomes. Lipid peroxidation was induced by adding 100 *μ*L of ferric nitrate (20 mM) and 100 *μ*L of ascorbic acid (100 mM). After incubation for 1 hr at 37°C, the reaction was stopped by the addition of 1 mL of TCA (10%) and 1 mL of (1.67%) TBA was added and the reaction mixture was boiled for 15 min, cooled, and centrifuged and the absorbance of the supernatant was measured at 532 nm.

### 2.8. Hydroxyl Radical Scavenging Assay

Hydroxyl radical scavenging activity was measured by the ability of the different concentrations of* Arnebia benthamii* extract to scavenge the hydroxyl radicals generated by the Fe^3+^-ascorbate-H_2_O_2_ system (Fenton reaction) [8]. The reaction mixture contained; 500 *μ*L of 2-deoxyribose (2.8 mM) in phosphate buffer (50 mM, pH 7.4), 200 *μ*L of premixed ferric chloride (100 mM), 100 *μ*L of H_2_O_2_ (200 mM) with or without the extract solution (100–500 *μ*g/mL). The reaction was triggered by adding 100 *μ*L of 300 mM ascorbate and incubated for 1 h at 37°C. 0.5 mL of the reaction mixture was added to 1 mL of TCA (10%), then 1 mL of 1% TBA was added to the reaction mixture. The mixture was heated for 15 min on a boiling water bath. After the mixture being cooled, the absorbance at 532 nm was noted against a blank (the same solution but without reagent). The scavenging activity on hydroxyl radical was calculated as follows:

(2) Scavenging activity(%) =(1−absorbance  of  sampleabsorbance  of  control)×100.

### 2.9. Antioxidant Activity against Oxidative Damage to DNA

Hydroxyl radicals generated by Fenton reaction were used to induce oxidative damage to DNA [9]. The reaction mixture (15 *μ*L) contained 25 mg of calf thymus DNA in 20 mM phosphate buffer saline (pH 7.4) and different concentrations of plant extract (10, 30, 50 and 80 *μ*g) were added and incubated with DNA for 15 min at room temperature. The oxidation was induced by treating DNA with 20 mM ferric nitrate and 100 mM ascorbic acid and incubated them for 1 h at 37°C. The reaction was terminated by the addition of loading buffer bromophenol blue (0.25%) and glycerol (30%) and the mixture was subjected to gel electrophoresis in 0.7% agarose/TAE buffer run at 100 V. DNA was visualized and photographed by gel doc.

### 2.10. Cell Lines and Culture

Human cancer cell lines lung (HOP-62, A549), prostate (PC-3), leukemia (THP-1), colon (HCT-116), and pancreatic (MIA-Pa-Ca) were obtained from IIIM Jammu. These cell lines were grown and maintained in a high glucose concentration (4.5 g/L) Dulbecco's modified Eagle medium supplemented with 10% fetal bovine serum and 1% penicillin-streptomycin (100 IU–100 mg/mL) in a humidified incubator at 37°C and in 5% CO_2_ atmosphere.

### 2.11. Cytotoxicity Assay

This assay was carried out as described by Sun et al. [10]. SRB assay is a rapid, sensitive, and inexpensive method for measuring the cytotoxic potential of test substances, based on the cellular protein content of adhered suspension cultures in 96 well plates. This method is suitable for ordinary laboratory purposes and for large-scale applications like high through put* in vitro* screening in anticancer drug discovery. The anticancer activity was determined by the cytotoxic potential of the test material using human cancer cell line, which was allowed to grow on tissue culture plate in the presence of test material. The cell growth was measured on ELISA reader after staining with Sulphorhodamine B (SRB) dye which binds to basic amino acid residues in trichloroacetic acid (TCA) fixed cells.

### 2.12. Statistical Analysis

The values are expressed as mean ± standard deviation (SD). The results were evaluated by using the SPSS (version 12.0) and Origin 6 software and evaluated by one-way ANOVA. The IC_50_ values were calculated by using Origin 6.0 version by plotting the percentage inhibition versus the concentrations. The quality of the radical scavenging property of the extracts was determined by calculating the IC_50_. The IC_50_ value is the concentration of each extract required to scavenge the free radical to 50% of the control.

## 3. Results and Discussion

### 3.1. Total Phenolic Content

**Figure 2:**
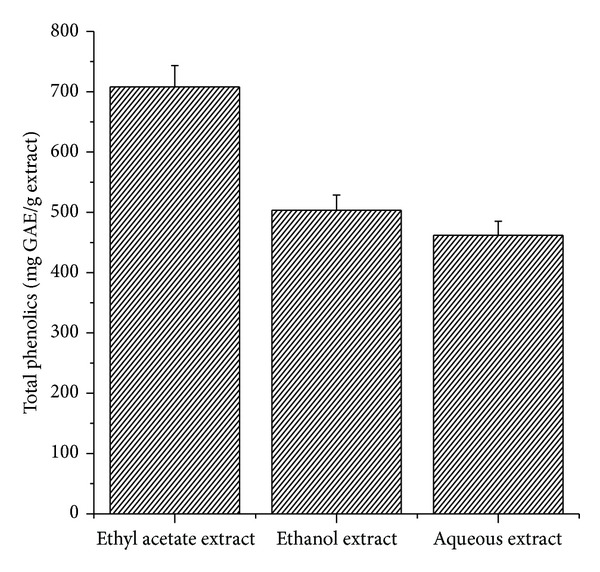
Total phenolic content of ethyl acetate, ethanol, and aqueous extracts of* Arnebia benthamii*. Data are presented as the mean value ± standard deviation of 3 separate experiments.

Phenolic compounds in plants are powerful free radical scavengers that can inhibit lipid peroxidation by neutralizing peroxyl radicals generated during the oxidation of lipids [11]. The TPC of the different extracts of* Arnebia benthamii* was assayed by the Folin-Ciocalteu method using gallic acid as standard. It was found that the TPC of different extracts was in the descending order of ethyl acetate > ethanol extract > aqueous extract (Figure 2). The highest TPC of 780 mg GAE/g was obtained in ethyl acetate, whereas the lowest TPC of 462 mg GAE/g was achieved in aqueous extract. It is worthwhile to mention that the TPC of ethanol extract was lower than that of ethyl acetate extract, but higher than that of aqueous extract, which may be the result of enrichment of the phenolic components in the extracts.

### 3.2. DPPH Radical Scavenging Activity

**Figure 3:**
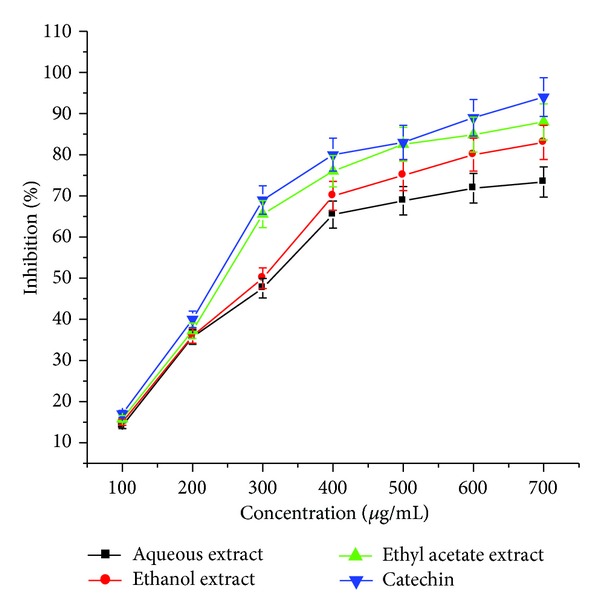
DPPH radical scavenging activity of aqueous, ethyl acetate, and ethanol crude extract* Arnebia benthamii*. Data are presented as the mean value ± standard deviation of 3 separate experiments. Absorbance at 517 nm.

DPPH radical scavenging assay is one of the most commonly used methods to evaluate the radical scavenging activity of antioxidants because of its quickness, reliability, and reproducibility. This method depends on the reduction of the purple DPPH by accepting an electron or hydrogen radical to become a stable diamagnetic molecule with discoloration. The degree of discoloration indicates the free radical scavenging potentials of the antioxidant compounds or extracts in terms of hydrogen-donating ability [12, 13]. DPPH free radical scavenging activities of the different extracts of* Arnebia benthamii* are shown in Figure 3. For each sample, seven concentrations (100–700 *μ*g/mL) of the plant extract were tested. All tested extracts showed a promising DPPH scavenging effect in a concentration-dependent manner. Ethyl acetate extract exhibited considerably higher DPPH radical scavenging activity than other two extracts, and the lowest DPPH radical scavenging rate was found in aqueous extract. The free radical scavenging activities of different extracts decreased in the order of ethyl acetate extract > ethanol extract > aqueous extract. The DPPH radical scavenging activity of these extracts positively correlated with the total phenolic content. The results are considered to be noteworthy when compared to our previous findings that ethyl acetate extract of* Podophyllum hexandrum* rhizome showed a maximum percentage inhibition of 85.77% on DPPH [14]. The IC_50_ values were also calculated to further evaluate the antioxidant activity, as shown in Table 1. The lower the IC_50_ value is, the greater the free radical scavenging activity is. The highest DPPH radical scavenging effect was obtained in ethyl acetate extract with the lowest IC_50_ of 250 *μ*g/mL, followed by ethanol extract (300 *μ*g/mL) and aqueous extract (335 *μ*g/mL). As we have taken catechin as a standard, it showed higher radical scavenging ability with IC_50_ of 230 *μ*g/mL once compared with different extracts. Results of our study suggest that the plant extracts with higher concentration of phytochemical constituents have increased capability of donating hydrogen atom to scavenging free radicals.

### 3.3. Reducing Power

**Figure 4:**
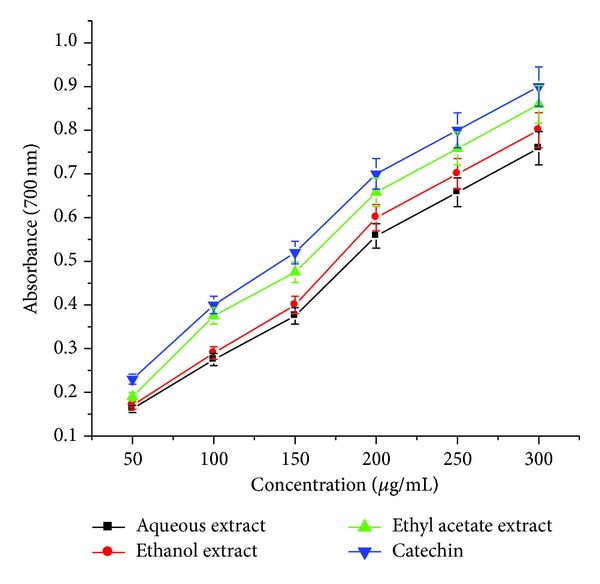
Reducing power of aqueous, ethyl acetate, and ethanol crude extracts* Arnebia benthamii*. Data are presented as the mean value ± standard deviation of 3 separate experiments. Absorbance at 700 nm.

In reducing power assay, potential antioxidants reduce the Fe^3+^/ferricyanide complex to its ferrous form which can then be monitored spectrophotometrically at 700 nm. Increased absorbance of the reaction mixture indicates increased reducing power. The antioxidant activities of natural components may have a reciprocal correlation with their reducing powers.  Figure 4 shows the dose-response curves for the reducing power of all the extracts of* Arnebia benthamii.* The reducing power values were found to be correlated with the concentration of each extract. The highest reducing power among the extracts was found in ethyl acetate extract, followed by ethanol and aqueous extract. Significantly higher reducing power (0.859) was observed for ethyl acetate extract at 300 *μ*g/mL, while as it was 0.802, 0.759, and 0.901 for ethanol extract, aqueous extrac,t and catechin, respectively. In our earlier studies, we observed similar results with aqueous extracts of* Podophyllum hexandrum* that the reducing power activity increased with the increase in the extract concentration [15].

### 3.4. Lipid Peroxidation

Lipid peroxidation in biological systems has long been thought to be a toxicological phenomenon that can lead to various pathological consequences [16]. Fe^2+^-ascorbic acid mixture is well known to stimulate lipid peroxidation in rat liver* in vivo* and in microsomes and mitochondria of rat liver* in vitro *[17]. Since it is believed that lipid peroxidation is one of the causes of the occurrence of cardiovascular disease [18] and cancer [19], its high inhibition by extracts of plants may represent an indicator of their high therapeutic potential. Furthermore, it has been shown that flavonoids have the capacity to terminate the chain reaction of lipid peroxidation by scavenging the peroxyl radical LOH [20].

**Figure 5:**
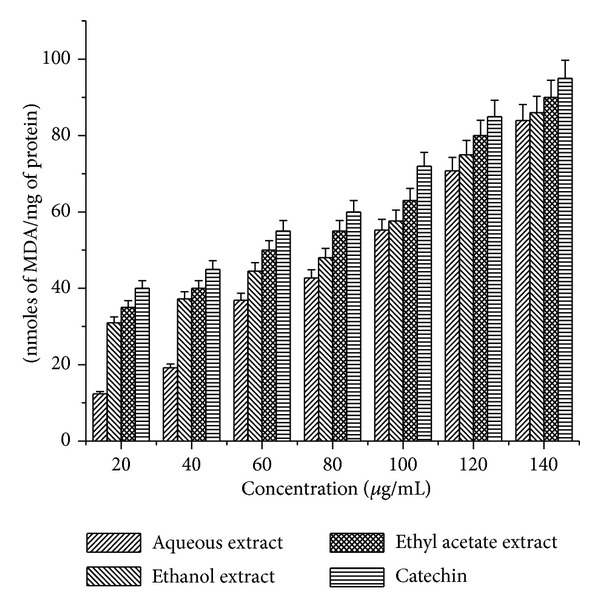
Microsomal lipid peroxidation of aqueous, ethyl acetate, and ethanol crude extracts* Arnebia benthamii*. Data are presented as the mean value ± standard deviation of 3 separate experiments. Absorbance at 532 nm.

The inhibitory effect of* Arnebia benthamii* extracts and catechin on TBARS production in rat liver microsomes induced by ferric nitrate-ascorbic acid/H_2_O_2_ is shown in Figure 5. Results showed that the inhibition of TBARS formation increased with increasing concentrations of* Arnebia benthamii* extracts and catechin. At concentrations of 20–140 *μ*g/mL, all the three extracts displayed a different potency of antilipid peroxidation activity, with an inhibition rate for aqueous extract that varies from 12.35% to 83.94%; for ethanol extract the inhibition rate was found to be 30.98% to 86.10 and for ethyl acetate extract the percentage inhibition varies from 35% to 95%, respectively. The IC_50_ values were also calculated as shown in Table 1. The highest antilipid peroxidation effect was again obtained in ethyl acetate extract with the lowest IC_50_ of 60 *μ*g/mL, followed by ethanol extract (82 *μ*g/mL) and aqueous extract (85 *μ*g/mL).

### 3.5. Hydroxyl Radical Scavenging Activity Radical

**Figure 6:**
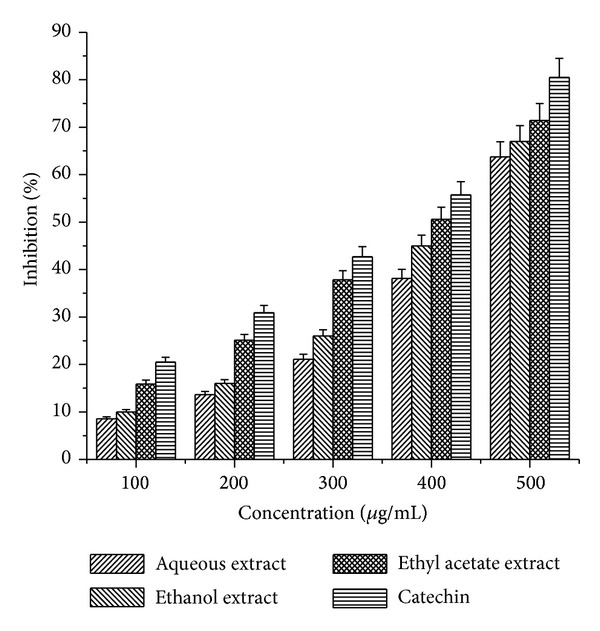
Hydroxyl radical scavenging activity of aqueous, ethyl acetate, and ethanol crude extracts* Arnebia benthamii*. The results represent mean ± S.D of 3 separate experiments. Results are reported as the percentage of the maximum formation of OH^*∙*^ radical (100% deoxyribose oxidized): in absorbency, 100% is 1.270 ± 0.007 (control). Absorbance at 532 nm.

The hydroxyl radical is an extremely reactive free radical formed in biological systems and has been implicated as a highly damaging species in free radical pathology, capable of damaging almost every molecule found in living cells [16]. This radical has the capacity to react with nucleotides of DNA and cause strand breakage, which leads to carcinogenesis, mutagenesis, and cytotoxicity. In addition, this species is considered to be one of the quick initiators of the lipid peroxidation process, abstracting hydrogen atoms from unsaturated fatty acids. The hydroxyl radical scavenging activity of the three different extracts of* Arnebia benthamii* at the concentration range of (100–500 *μ*g/mL) can be ranked as ethyl acetate > ethanol > aqueous extract (Figure 6). All the extracts exhibited good hydroxyl radical scavenging activity with 63.75% for aqueous extract, 67% for ethanol extract, and 71.42% for ethyl acetate extract at the highest concentration used (500 *μ*g/mL). The ability of the above mentioned extracts to quench hydroxyl radicals seems to be directly related to the prevention of propagation of the process of lipid peroxidation and seems to be good scavenger of reactive oxygen species.

### 3.6. Antioxidant Activity against Oxidative Damage to DNA

**Figure 7:**
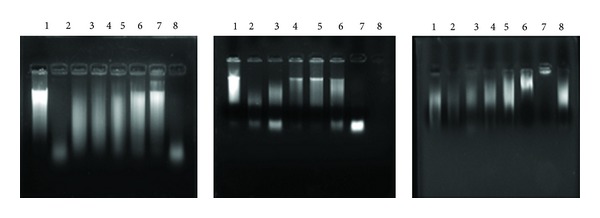
Protective effect of ethyl acetate, ethanol, and aqueous extracts of* Arnebia benthamii* on oxidative damage to calf thymus DNA. Lane 1: native calf thymus DNA, lane 2: DNA + 20 mM ferric nitrate + 100 mM ascorbic acid + 30 mM H_2_O_2_, lane 3: DNA + 20 mM ferric nitrate + 100 mM ascorbic acid + 30 mM H_2_O_2_ + 10 *μ*g of plant extract, lane 4: DNA + 20 mM ferric nitrate + 100 mM ascorbic acid + 30 mM H_2_O_2_ + 20 *μ*g of plant extract, lane 5: DNA + 20 mM ferric nitrate + 100 mM ascorbic acid + 30 mM H_2_O_2_ + 30 *μ*g of plant extract, lane 6: DNA + 20 mM ferric nitrate + 100 mM ascorbic Acid + 30 mM H_2_O_2_ + 50 *μ*g of plant extract, lane 7: DNA + 20 mM ferric nitrate + 100 mM ascorbic acid + 30 mM H_2_O_2_ + 80 *μ*g of plant extract, and lane 8: DNA + 20 mM ferric nitrate + 100 mM ascorbic acid + 30 mM H_2_O_2_ + 10 *μ*g of catechin.

The protective effect of* Arnebia benthamii* extracts on calf thymus DNA is shown in the Figure 7. Hydroxyl radicals generated by Fenton reaction were found to induce DNA strand breaks in calf thymus DNA. H_2_O_2_ alone did not cause DNA strand cleavage. However in presence of ferric nitrate and ascorbic acid, H_2_O_2_ leads to high DNA damage (lane 20).* Arnebia benthamii* extracts at 10–80 *μ*g offered complete protection to DNA damage induced by hydroxyl radicals in calf thymus DNA (lanes 3–7). Again our results indicate that ethyl acetate extracts showed strong DNA damage protection once compared with that of ethanol and aqueous extract (Figure 7). Thus, the hydroxyl radical quenching ability of polyphenolic compounds of* Arnebia benthamii* could be responsible for the protection against oxidative damage to DNA.

### 3.7. Cytotoxic Activity

The growth inhibitory effects of different solvent extracts of* Arnebia benthamii* on six human cancer cell lines (HOP-62, A549, PC-3, THP-1, HCT-116, and MIA-Pa-Ca) were tested with the SRB assay. Cells were treated with various concentrations (10–100 *μ*g/mL) of the extracts for 48 h. All the extracts did not exhibit significant effect on cells viability at the concentration of 10 *μ*g/mL. However, at the concentrations of 50 and 100 *μ*g/mL the extracts inhibited cell proliferation in a concentration-dependent manner on most of the cell lines, Table 2. Among those cancer cells tested, HOP-62, A549, THP-1, and MIA-Pa-Ca were the most sensitive cancer cells when treated with methanol extract of* Arnebia benthamii *with percentage inhibition of 100, 100, 90, and 100% at the concentration of 100 *μ*g/mL, Table 2. The most resistant cancer cell to the extracts-induced growth inhibition was found to be PC-3 (prostrate) with 0% for methanol, aqueous, and ethanol extracts, 35% with ethyl acetate extract, and 39% with petroleum ether extract at the concentration of 100 *μ*g/mL. As shown in Table 2, ethyl acetate extract comparably showed stronger growth inhibition on all the cell lines but less than the methanolic extract at the 100 *μ*g/mL, indicating that the active anticancer compounds were mainly concentrated in the methanol and ethyl acetate extracts of* Arnebia benthamii*. Interestingly, the antiproliferation effect of methanolic extract was higher on HOP-62 and A549 than the known anticancer drug Paclitaxel. Ethyl acetate extract showed comparable inhibition on THP-1, MIA-Pa-ca, and HCT-116 cell lines. Similar results were observed in our previous study, with the 70% ethanolic and methanolic extracts of* Podophyllum hexandrum*, where both the extracts showed strong anticancer activities against different human cancer cells [21].

## 4. Conclusion

The results of the present study provide an evidence that antioxidant properties of* Arnebia benthamii* extracts showed mainly the ethyl acetate and ethanol extracts to be the potent source of antioxidants which positively correlates with their total phenolic content. Furthermore, the ethyl acetate and methanol extracts also showed the potent cytotoxic activity on six human cancer cell lines. Therefore,* Arnebia benthamii* extracts especially ethyl acetate, methanol, and ethanol deserves further investigation in active compounds responsible for the antioxidant and anticancer properties as it might be used in the field of pharmaceutical products and functional foods for the preservation and treatment of cancers.

## Figures and Tables

**Table 1:** Antioxidant assays (IC_50_ values) of different extracts of *Arnebia  benthamii*.

Extracts	IC_50_/DPPH	IC_50_/reducing power	IC_50_/lipid peroxidation	IC_50_/DNA damage
Aqueous extract	335	195	85	415
Ethanol extract	300	185	82	425
Ethyl acetate extract	250	165	60	400
Catechin	230	145	45	325

**Table 2:** Cytotoxicity of the different crude extracts of *Arnebia  benthamii* and 5-fluorouracil (5-FU) and Paclitaxel on six human cancer cell lines.

Cell line type			HOP-62	A549	PC-3	THP-1	HCT-116	MIA-Pa-Ca
Tissue type			Lung	Lung	Prostate	Leukemia	Colon	Pancreatic
S. no.	Code	Conc. (*μ*g/mL)	%Age growth inhibition					
1	EB-MET	100	100	100	0	90	63	100
2	EB-MET	50	46	13	0	45	49	47
3	EB-MET	10	33	18	0	48	40	25
4	EB-ETH	100	55	42	0	65	57	55
5	EB-ETH	50	49	24	0	62	57	44
6	EB-ETH	10	43	0	0	57	47	40
7	EB-EA	100	39	21	35	68	72	66
8	EB-EA	50	24	0	6	7	8	23
9	EB-EA	10	0	0	0	0	0	0
10	EB-PE	100	47	54	39	29	19	0
11	EB-PE	50	47	53	10	18	14	0
12	EB-PE	10	39	15	0	1	12	0
13	EB-AQ	100	35	36	0	56	44	40
14	EB-AQ	50	32	24	0	28	42	30
15	EB-AQ	10	24	17	0	9	10	12
16	5-FU	20 *μ*M	—	—	—	**67**	**67**	—
17	Paclitaxel	1 *μ*M	**72**	**70**	—		—	—

Data are means ± SD of three independent experiments.

## References

[1] Fang Y, Yang S, Wu G (2002). Free radicals, antioxidants, and nutrition. *Nutrition*.

[2] Anderson D (1999). Antioxidant defences against reactive oxygen species causing genetic and other damage. *Mutation Research*.

[3] Sun B, Fukuhara M (1997). Effects of co-administration of butylated hydroxytoluene, butylated hydroxyanisole and flavonoids on the activation of mutagens and drug-metabolizing enzymes in mice. *Toxicology*.

[4] Nurmi K, Ossipov V, Haukioja E, Pihlaja K (1996). Variation of total phenolic content and individual low-molecular-weight phenolics in foliage of mountain birch trees (*Betula pubescens* ssp. tortuosa). *Journal of Chemical Ecology*.

[5] Kim D, Lee KW, Lee HJ, Lee CY (2002). Vitamin C equivalent antioxidant capacity (VCEAC) of phenolic phytochemicals. *Journal of Agricultural and Food Chemistry*.

[6] Oyaizu M (1978). Studies on products of browning reaction—antioxidant activities of products of browning reaction prepared from glucosamine. *Japanese Journal of Nutrition*.

[7] Urata Y, Yoshida S, Irie Y (2002). Treatment of asthma patients with herbal medicine TJ-96: a randomized controlled trial. *Respiratory Medicine*.

[8] Ilavarasan R, Mallika M, Venkataraman S (2005). Anti-inflammation and antioxidant activities of *Cassia fistula* Linn. bark extracts. *African Journal of Traditional, Complementary and Alternative Medicine*.

[9] Ani V, Varadaraj MC, Akhilender K (2006). Antioxidant and antibacterial activities of polyphenolic compounds from bitter cumin (*Cuminum nigrum* L.). *European Food Research and Technology*.

[10] Sun S, Yue P, Dawson MI (1997). Differential effects of synthetic nuclear retinoid receptor-selective retinoids on the growth of human non-small cell lung carcinoma cells. *Cancer Research*.

[11] Shahidi F, Wanasundara PK (1992). Phenolic antioxidants. *Critical Reviews in Food Science and Nutrition*.

[12] Alasalvar C, Karamać M, Kosińska A, Rybarczyk A, Shahidi F, Amarowicz R (2009). Antioxidant activity of hazelnut skin phenolics. *Journal of Agricultural and Food Chemistry*.

[13] Shahidi F, Alasalvar C, Liyana-Pathirana CM (2007). Antioxidant phytochemicals in hazelnut kernel (*Corylus avellana* L.) and hazelnut byproducts. *Journal of Agricultural and Food Chemistry*.

[14] Ganie SA, Haq E, Masood A, Hamid A, Zargar MA (2011). Antioxidant and protective effect of ethyl acetate extract of podophyllum hexandrum rhizome on carbon tetrachloride induced rat liver injury. *Evidence-Based Complementary and Alternative Medicine*.

[15] Showkat AG, Shajrul A, Rabia H (2012). Podophyllum hexandrum aqueous extract as a potential free radical scavenger. *Redox Report*.

[16] Hochstein P, Atallah AS (1988). The nature of oxidants and antioxidant systems in the inhibition of mutation and cancer. *Mutation Research*.

[17] Kornbrust DJ, Mavis RD (1980). The effect of paraquat on microsomal lipid peroxidation in vitro and in vivo. *Toxicology and Applied Pharmacology*.

[18] Halliwell B, Gutteridge JMC (1999). *Free Radicals in Biology and Medicineed*.

[19] Takabe W, Niki E, Uchida K, Yamada S, Satoh K, Noguchi N (2001). Oxidative stress promotes the development of transformation: involvement of a potent mutagenic lipid peroxidation product, acrolein. *Carcinogenesis*.

[20] Takahama U (1983). Redox reactions between kaempferol and illuminated chloroplasts. *Plant Physiology*.

[21] Ganie SA, Haq E, Hamid A, Masood A, Zargar MA (2011). Long dose exposure of hydrogen peroxide (H_2_O_2_) in albino rats and effect of *Podophyllum hexandrum* on oxidative stress. *European Review for Medical and Pharmacological Sciences*.

